# Need for ensuring care for neuro-emergencies—lessons learned from the COVID-19 pandemic

**DOI:** 10.1007/s00701-020-04437-z

**Published:** 2020-06-08

**Authors:** Nils Hecht, Lars Wessels, Finn-Ove Werft, Ulf C. Schneider, Marcus Czabanka, Peter Vajkoczy

**Affiliations:** Department of Neurosurgery and Center for Stroke Research Berlin (CSB), Charité - Universitätsmedizin Berlin, Charitéplatz 1, 10117 Berlin, Germany

**Keywords:** COVID-19 pandemic, Coronavirus, Collateral damage, Neurosurgery, Subarachnoid hemorrhage, Chronic subdural hematoma

## Abstract

**Background:**

To investigate whether patients with critical emergency conditions are seeking or receiving the medical care that they require, we characterized the reality of care for patients presenting with neuro-emergencies during the first phase of the COVID-19 pandemic.

**Methods:**

In this observational, longitudinal cohort study, all neurosurgical admissions that presented to our department between February 1 and April 15 during the COVID-19 pandemic and during the same time period in 2019 were identified and categorized according to the presence of a neuro-emergency, the route of admission, management, and the category of disease. Further, the clinical course of patients with aneurysmal subarachnoid hemorrhage (aSAH) and chronic subdural hematoma (cSDH) was investigated representatively for severe vascular and semi-urgent traumatic conditions that present with a wide variety of symptoms.

**Results:**

During the pandemic, the percentage of neuro-emergencies among all neurosurgical admissions remained similar but a larger proportion presented through the emergency department than through the outpatient clinic or by referral (*p = 0.009). The total number of neuro-emergencies was significantly reduced (*p = 0.0007) across all types of disease, particularly in vascular (*p = 0.036) but also in spinal (*p = 0.007) and hydrocephalus (*p = 0.048) emergencies. Patients with spinal emergencies presented 48 h later (*p = 0.001) despite comparable symptom severity. For aSAH, the number of cases, aSAH grade, aneurysm localization, and treatment modality did not change but strikingly, elderly patients with cSDH presented less frequently, with more severe symptoms (*p = 0.046), and were less likely to reach favorable outcome (*p = 0.003) at discharge compared with previous years.

**Conclusions:**

Despite pandemic-related restrictive measures and reallocation of resources, patients with neuro-emergencies should be encouraged to present regardless of the severity of symptoms because deferred presentation may result in adverse outcome. Thus, conservation of critical healthcare resources remains essential in spite of fighting COVID-19.

## Introduction

As the COVID-19 pandemic continues to develop, many countries have taken incisive measures to limit the spread of SARS-CoV-2 [18], such as a restriction of social and economic life. These measures, together with the fear of infection [12], have changed peoples’ routines in drastic fashion in a very short period of time [1]. Against this background, fear of COVID-19 may prevent patients with critical medical or surgical emergencies from actively presenting in emergency departments and outpatient clinics [2, 15]. Further, reallocation measures and triaging of medical services with the purpose to ensure care for a potential surge of COVID-19 patients represent an unprecedented challenge for all specialties that remain committed to medical emergencies that require immediate attention [5, 9, 10, 19]. National and international concerns in this direction have been voiced in the field of cardiology and neurology, where it is feared that patients with acute coronary heart syndrome or cerebral stroke might avoid hospitals and emergency rooms due to social distancing or fear of acquiring COVID-19 [8, 11]. Next to such acutely life-threatening emergencies, it seems even more conceivable that patients suffering milder symptoms may currently rather tolerate these than expose themselves to a potential risk of infection that may be associated with hospitalization. Here, neurosurgery offers the opportunity to investigate how the effects of the COVID-19 pandemic may have influenced non-elective emergency care, because neuro-emergencies occur with a wide spectrum of neurological symptoms and severity of disease. In this observational, longitudinal cohort study, we captured the development of neuro-emergencies in one of the largest European university hospitals in the first phase of the COVID-19 pandemic.

## Methods and materials

### Study design and patient population

This longitudinal cohort study was approved by the local ethics committee of the Charité University Hospital in Berlin, Germany (EA1/097/20), and is reported according to the STROBE statement (http://www.strobe-statement.org/). Informed consent was waived due to the retrospective nature of the study. To analyze the effect of the COVID-19 pandemic on neuro-emergency admissions, we identified all patients that were admitted to the Department of Neurosurgery at the Charité University Hospital in Berlin between February 1 and April 15 in the years 2019 and 2020. The time window was chosen based on the identification of the first COVID-19-positive patient in Germany on January 28, 2020, which is considered the beginning of the COVID-19 period when social life and medical operations in Germany were beginning to get affected. Social distancing in Germany was officially recommended by the federal government on March 12, 2020. Data review and analysis was performed by observers (NH, LW, and FW) who were not directly involved in the admission and triage process.

### Definition of neurosurgical emergencies

All patients treated during the pandemic were triaged according to a recently published consensus statement from the German Society of Neurosurgery (DGNC) and the Association of German Neurosurgeons (BDNC) regarding the definition of non-elective cases (https://www.dgnc.de/gesellschaft/aktuelles/statements). After an individual review of the patients’ charts and neuroimaging, neuro-emergencies were assigned to 1 of 6 disease categories. Specifically, these included the following: *vascular* (aneurysmal subarachnoid hemorrhage (aSAH), malignant cerebral infarction, space-occupying intracerebral hematoma, hemorrhage due to arteriovenous (AV) malformations, higher grade dural AV fistulas, procedures including revascularization in patients with evidence of relevant vascular occlusive disease and unstable aneurysms), *cranial oncological* (malignant primary brain tumors, brain metastases of any primary tumor type, benign or low-grade tumors with marked parenchymal compression or progressive neurological deficits and pituitary tumors with cranial nerve deficits, visual impairment or endocrine deficiency that cannot be managed conservatively), *spinal* (intraspinal pathologies with signs of spinal cord compression, degenerative spine conditions with acute onset of motor deficits and/or vegetative dysfunction, progressive myelopathy of cervical and/or thoracic spine, vertebral body fractures with therapy-refractory and severe pain, instability and/or signs of spinal cord compression and spinal metastases or primary tumors with therapy-refractory severe pain, instability and/or compression of the spinal cord), *traumatic brain injury* (acute traumatic brain injury with subdural hematoma and/or epidural hematoma, any scenario where intracranial pressure cannot be controlled by means of conservative management and chronic subdural hematoma with neurological symptoms), *hydrocephalus* (progressive increase of intracranial pressure with signs and symptoms suggestive of elevated intracranial pressure or shunt dysfunction), *infection*, or *other* emergencies (pain syndromes that do not respond to non-invasive therapeutic modalities, battery depletion in DBS and SCS patients, and benign or malignant peripheral nerve tumors with neurological deficits). The development of emergency admissions was then estimated by calculating the stepwise increase of emergency admissions for each of the six disease categories during the pre-specified time window in 2019 and 2020.

### Neurosurgical measures during the COVID-19 pandemic

To maintain a fully operational neurosurgical service, on March 12, 2020, our department implemented pre-specified cohort formation (faculty and residents) with definition of clean cohorts at each campus, home-office, distancing and hygiene measures with outpatient re-organization to video- or telephone appointments, video conference calls for all clinical and educational purposes, business trip ban, and voluntary limitation of social interaction outside of the workplace. For all admitted patients, COVID-19 diagnostics were executed in case of a body temperature > 37.3 °C, a quick Sepsis-related Organ Failure Assessment (qSOFA) [16] score ≥ 1, in the presence of respiratory symptoms, and/or in the case of previous contact to another person with confirmed COVID-19. The protocol included three repetitive, combined deep bilateral nasal and deep oropharyngeal swabs for detection of SARS-CoV-2 RNA through PCR analysis, phlegm analysis for SARS-CoV-2 and/or bacterial infection, and chest X-ray.

### Statistical analysis

Descriptive summary statistics are presented as median and range or percentage, as appropriate. Normality was determined with the Shapiro-Wilk test. Statistics were calculated with GraphPad Prism for Mac (Version 8.1.0, GraphPad Software, San Diego, CA, USA). For contingency analysis, a chi-square test was used. For comparison of age in both populations, a two-tailed *t* test was performed. For comparison of the stepwise increase of neuro-emergency admissions for 2019 and 2020, a Wilcoxon matched-pairs signed-rank test was used. For patients with chronic subdural hematoma (cSDH), the stepwise increase of cases between 2014 and 2019 was compared with that of 2020 with a Friedman test for matched pairs and uncorrected Dunn’s test for multiple comparison. For comparison of symptom duration, a Mann-Whitney test was performed. All tests were two-tailed and statistical significance was set at *p* < 0.05.

## Results

### Patient characteristics

Demographic and clinical data are presented in Table 1. Between February 1 and April 15, 2020, the number of overall admissions to our department was 46% lower than during the same time period of the previous year. Also, a significantly higher percentage of emergency room admissions was noted (76% versus 61%) at the cost of outpatient admissions and referrals compared with 2019 (*p = 0.009). Twenty-one out of 352 admissions in 2020 received COVID-19 PCR diagnostics following hospitalization. None had evidence of SARS-CoV-2 RNA.

**Table 1:** Demographics and clinical presentation

	2019	2020	
Total number of admissions (*n*)	655	352	
Age in years (median, range)	60 (19–94)	59 (19–92)	*p* = 0.255
Age group in years, *n* (%)			*p* = 0.424
≤ 70	455 (69%)	253 (72%)	
> 70	200 (31%)	99 (28%)	
Gender (*n*, %)			*p* = 0.618
Male	326 (50%)	181 (51%)	
Female	329 (50%)	171 (49%)	
Type of admission (*n*, %)			*p* = 0.226
Emergency	217 (33%)	130 (37%)	
Non-emergency	438 (67%)	222 (63%)	
Admission route of emergencies (*n*, %)			*p = 0.009
Emergency room	132 (61%)	99 (76%)	
Outpatient clinic	18 (8%)	4 (3%)	
External referral	67 (31%)	27 (21%)	
Category of emergency admissions (*n*, %)			*p* = 0.328
Vascular	39 (18%)	18 (14%)	
Cranial oncological	40 (18%)	28 (22%)	
Spinal	43 (20%)	21 (16%)	
Trauma	51 (24%)	41 (31%)	
Hydrocephalus	14 (6%)	5 (4%)	
Infection	25 (12%)	11 (8%)	
Other	5 (2%)	6 (5%)	
Management of emergencies (*n*, %)			*p* = 0.665
Surgical	127 (59%)	73 (56%)	
Non-surgical	90 (41%)	57 (44%)	

### Presentation and clinical course of neuro-emergencies

**Fig. 1:**
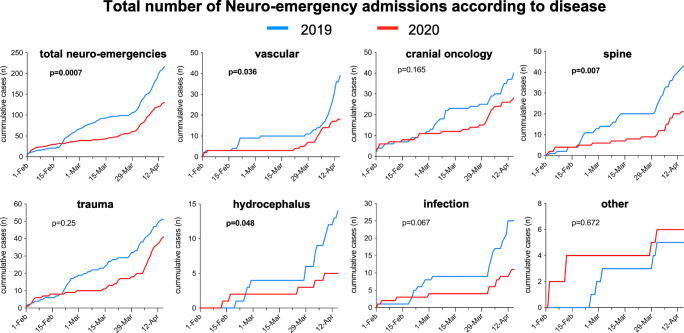
Line graphs of the total and individual types of neuro-emergencies that were admitted to our department between February 1 and April 15 in 2019 (blue) compared with the same time period during the COVID-19 pandemic in 2020 (red). During the pandemic, a significantly lower number of admissions with neuro-emergencies (*p = 0.0007) and specifically vascular (*p = 0.036), spinal (*p = 0.007), and hydrocephalus emergencies (*p = 0.048) were noted compared with the same time period in 2019

Although no significant difference was noted in the frequency distribution of neuro-emergency admissions during the pandemic (Table 1), a significant (40%) reduction in the total number of neuro-emergency admissions was noted across all types of diseases (*p = 0.0007; Fig. 1) and particularly for vascular (*p = 0.036), spinal (*p = 0.007), and hydrocephalus (*p = 0.048) emergencies (Fig. 1). To get a better idea of the of the pattern of seeking emergency care, we analyzed the frequency distribution of symptoms according to severity for spinal, vascular, hydrocephalus, and trauma emergencies and detected no difference in the distribution of the symptom severity (Table 2). Regarding the time from symptom onset to presentation, however, patients with spinal emergencies had a significantly longer duration of symptoms (median 72 h, IQR 18–84) than in 2019 (median 24 h, IQR 9–48; *p = 0.001) (Table 2).

**Table 2:** Duration and severity of symptoms. *IQR*, interquartile range; *GCS*, Glasgow Coma Scale score

	2019	2020	
Hours from symptom onset to presentation (median, IQR)			
Spine	24 (9–48)	72 (18–84)	*p = 0.001
Vascular emergency	4 (3–8)	6 (3–36)	*p* = 0.53
Hydrocephalus	3 (1–24)	4 (2–48)	*p* = 0.67
Traumatic brain injury	1 (1–1)	1 (1–2)	*p* = 0.48
Symptoms—spine (*n*, %)			*p* = 0.29
Asymptomatic	-	-	
Pain	27 (63%)	10 (48%)	
Focal neurological deficit	16 (37%)	11 (52%)	
Reduced GCS	-	-	
Symptoms—vascular emergency (*n*, %)			*p* = 0.61
Asymptomatic	-	-	
Pain	13 (33%)	4 (22%)	
Focal neurological deficit	17 (44%)	8 (44%)	
Reduced GCS	9 (23%)	6 (34%)	
Symptoms—hydrocephalus (*n*, %)			*p* = 0.91
Asymptomatic	2 (14%)	1 (20%)	
Pain	5 (36%)	1 (20%)	
Focal neurological deficit	3 (21%)	1 (20%)	
Reduced GCS	4 (29%)	2 (40%)	
Symptoms—traumatic brain injury (*n*, %)			*p* = 0.48
Asymptomatic	15 (29%)	7 (17%)	
Pain	10 (20%)	10 (24%)	
Focal neurological deficit	9 (18%)	6 (15%)	
Reduced GCS	17 (33%)	18 (44%)	

**Fig. 2:**
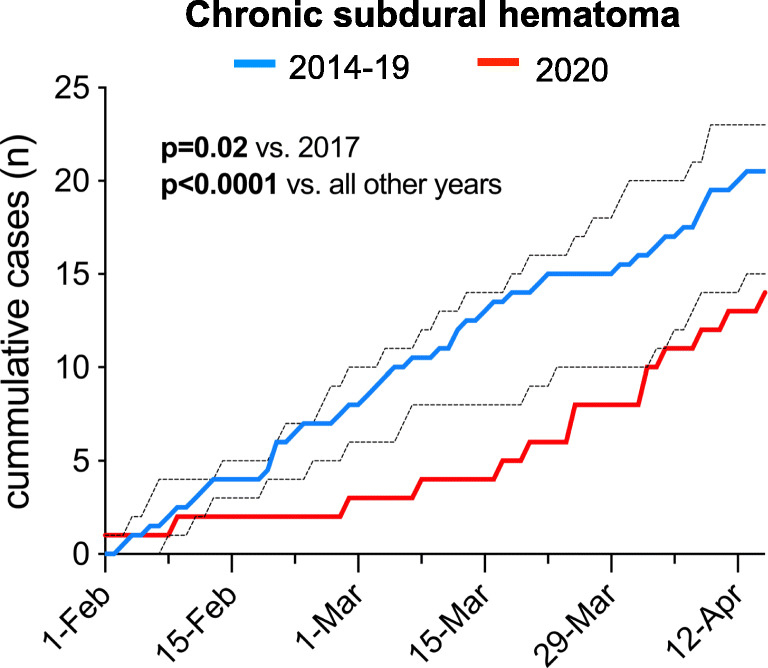
Line graph illustrating the reality of care for emergency admissions and treatment of chronic subdural hematoma (cSDH) during the COVID-19 pandemic between February 1 and April 15 compared with the same time period of the years 2014–2019. For 2014–2019, data is presented as median and the dotted lines illustrate the range (minimum to maximum). The day-by-day admission and treatment numbers during the pandemic were significantly lower compared with those in all previous years

For a more detailed description of the presentation and treatment indication of a *severe* neuro-emergency, we analyzed patients suffering aSAH and found that the number of cases, aSAH severity, aneurysm localization, and treatment modality had not changed during the pandemic (Table 3). Next, we analyzed patients suffering cSDH representative of a *mild-to-moderate* neuro-emergency, because cSDH can manifest with the entire range of neurological symptoms. Second, the trauma leading to cSDH most likely occurred before or at the beginning of the socio-economic restrictive measures, so that the incidence of cSDH should remain uninfluenced by pandemic-associated lifestyle changes. Third, cSDH has a high prevalence in the elderly [13] who are predisposed for an adverse course of COVID-19 [17]. Compared with previous years, significantly less patients received acute treatment for cSDH (*p = 0.02 for 2020 versus 2017 and *p < 0.0001 for 2020 versus any other year; Fig. 2) and strikingly, these patients significantly more often suffered severe than mild-to-moderate symptoms compared with previous years (*p = 0.046 for 2014–2019 versus 2020; Table 4). Further, cSDH patients during the pandemic had a significantly lower likelihood of experiencing favorable outcome at discharge (*p = 0.003 for mRS 0–2 in 2014–2019 versus 2020; Table 4).

**Table 3:** Presentation of patients suffering aneurysmal subarachnoid hemorrhage

	2019	2020	
Aneurysmal subarachnoid hemorrhage (*n*)	9	8	
Hunt and Hess grade (*n*, %)			*p* = 0.83
I	3 (33%)	3 (39%)	
II	2 (23%)	1 (12%)	
III	1 (11%)	1 (12%)	
IV	-	1 (12%)	
V	3 (33%)	2 (25%)	
Aneurysm localization (*n*, %)			*p* = 0.99
Anterior circulation	7 (78%)	6 (75%)	
Posterior circulation	2 (22%)	2 (25%)	
Treatment modality (*n*, %)			*p* = 0.99
Microsurgical	5 (56%)	4 (50%)	
Endovascular	4 (44%)	4 (50%)

**Table 4:** Presentation and outcome of patients suffering chronic subdural hematoma. *GCS*, Glasgow Coma Scale score; *mRS*, modified Rankin Scale score

	2014–2019	2020	
Age in years (median, range)	77 (33–95)	75 (46–85)	*p* = 0.285
Age group in years, *n* (%)			*p* = 0.178
≤ 70	31 (26%)	6 (43%)	
> 70	89 (74%)	8 (57%)	
Gender (*n*, %)			*p* = 0.59
Male	77 (64%)	10 (71%)	
Female	43 (36%)	4 (29%)	
Side (*n*, %)			*p* = 0.259
Left	53 (44%)	3 (21%)	
Right	41 (34%)	7 (50%)	
Bilateral	26 (22%)	4 (29%)	
Category of symptoms (*n*, %)			*p = 0.046
Headache/vertigo	50 (41%)	2 (14%)	
Motor deficit	38 (32%)	5 (36%)	
Reduced GCS	21 (18%)	4 (29%)	
Aphasia	11 (9%)	3 (21%)	
Outcome at discharge			*p = 0.003
mRS 0–2	95 (79%)	6 (43%)	
mRS 3–6	25 (21%)	8 (57%)	

## Discussion

In this study, we show that despite the restrictive measures associated with COVID-19, patients with neuro-emergencies continue to present across the entire spectrum of disease, which highlights the importance of maintaining a fully operational (neurosurgical) service in spite of reallocation of medical resources, facilities, and staff. For the first time, our findings suggest that patients who suffer a common, semi-urgent neuro-emergency and belong to a population at risk might defer immediate presentation and risk collateral damage.

On February 1, 2020, zero COVID-19 case was registered in Berlin among 16 cases in Germany. On March 12, 2020, social distancing was officially recommended by our federal government. On April 15, the number of COVID-19 infections in Berlin had exponentially increased to 4722 among 127,584 cases in Germany (https://corona.rki.de/). The Department of Neurosurgery at the Charité with its three sites serves a catchment area of approximately 3.7 million people, for whom emergency care may currently be hampered by constraints on facilities, staffing, and protective equipment. Further, COVID-19-related collateral effects may also occur due to deferral of systemically relevant care for medical emergencies, because patients may actively avoid emergency departments due to social-economic lockdown measures and fear of infection [2, 15]. This concern is supported by the fact that high-volume cardiac catheterization centers in the USA and Europe are experiencing a decreased hospitalization and catheterization rate of acute coronary syndrome and myocardial infarction [3, 6, 14]. In our study, we observed a similar development in the category of vascular emergencies. However, the presentation, severity, and treatment of potentially life-threatening aSAH remained unchanged. On the one hand, this reflects that we did not change our indication or perform hidden rationing or ethical triage for treatment of aSAH [9]. On the other hand, the development of aSAH shows that patients with acute aSAH continued to seek emergency medical care regardless of COVID-19, which stands in contrast to previous concerns and needs to be considered when developing strategies for resource reallocation and ethical triage [7, 9, 19]. Under the reasonable assumptions that the incidence of neuro-emergencies did not change during the first phase of the pandemic and that we experienced no access restriction to emergency medical care as other countries [4, 5, 19], the overall decline of vascular, spinal, and hydrocephalus emergencies rather suggests that less patients are currently seeking emergency medical care but *regardless* of the type and severity of disease [2, 15]. In spinal emergencies, this is supported by the delayed pattern of seeking care despite similar symptom severity as in 2019. Importantly, the collateral effect of such deferred presentation could also be mirrored by the fewer number of cSDH patients that presented with more severe symptoms and had a lower chance of favorable outcome, since cSDH represents a semi-urgent trauma emergency with high prevalence in patients most likely to defer an emergency presentation due to incapacity or fear of infection with SARS-CoV-2.

A natural limitation of our study is the single-center design, although from an epidemiological standpoint, we serve a well-defined catchment area as one of the highest volume neurosurgical centers in Europe. Of course, we cannot exclude that some patients might have presented at other hospitals, but for the majority of cases, this seems unlikely, because cranial neurosurgery in Germany is highly regionally focused. Alternatively, one might argue that we only managed to cope because we had sufficient resources and the pandemic did not hit us as hard compared with other regions in Europe, China, or the USA. However, a nation-wide reallocation of medical resources, facilities, and staff was implemented in the beginning of February 2020 in order to generate sufficient capacities for the expected surge of COVID-19 patients that may require isolation and/or intensive care. In mid-March 2020, we experienced a peak of this development as neurosurgical staff, OR staff, and nurses were reassigned to emergency departments and newly designated COVID-19 units, 25–30% of our regular hospital beds were blocked for COVID-19 patients, and our overall surgical capacity was reduced by 50%, which mirrors an international experience from other neurosurgical departments affected by COVID-19 [10]. For us, this resource reallocation translated into a reduction to 66 instead of 96 regular beds and overall, our hospital generated approximately 300 regular and intensive care unit (ICU) beds for COVID-19 patients. By the end of March, we experienced up to 75–80 COVID-19 patients requiring ICU care. The fact that patients even continued to present through our emergency department in an increased percentage and required surgery across the entire spectrum of disease underlines the importance of maintaining a fully functional service in spite of these pandemic-associated reallocation measures. Apart from cohort formation and containment, one of the most essential actions that ensured successful adjustment was an early commitment from our societies (DGNC and BDNC) to uphold care for specific non-elective neurosurgical procedures across the entire spectrum of disease. Also, we were able to maintain 15 out of 16 dedicated neurosurgical ICU beds across all of our sites. Another factor that helped us cope is that > 90% of our faculty and residents are generalists who are able to cover the entire neurosurgical spectrum rather than individual sub-specialists, so that the strain on our neurosurgical workforce by cohort measures, sick leave, and the ordered reduction of OR capacity was compensated by pushing the available OR resources to run 24 h a day, 7 days a week. Therefore, despite an extremely restrictive environment regarding OR capacities, staff, and facilities, we were able to maintain care for neuro-emergencies that also regularly received a high independent prioritization in the objective triage of OR resources.

In summary, our findings substantiate the following: First, patients with neuro-emergencies appear to be scared of COVID-19. Second, fear of COVID-19 resulted in a 40% reduction of neuro-emergency presentations, regardless of the disease or severity of symptoms. Third, patients that present despite COVID-19 require neurosurgical treatment, which underlines high systemic relevance and suggests that neurosurgery should be prioritized in the reallocation of critical healthcare resources. Ultimately, it will be essential to quantify collateral effects that may have resulted from deferred clinical presentation and we believe that our experience may help limit such effects in future situations requiring resource reallocation and triaging of medical services.
